# Influence of Resin Content and Density on Water Resistance of Bamboo Scrimber Composite from a Bonding Interface Structure Perspective

**DOI:** 10.3390/polym14091856

**Published:** 2022-04-30

**Authors:** Yaohui Ji, Wencheng Lei, Yuxiang Huang, Jiangyuan Wu, Wenji Yu

**Affiliations:** Research Institute of Wood Industry, Chinese Academy of Forestry, Beijing 100091, China; jiyaohui1994@gmail.com (Y.J.); leiwenc@163.com (W.L.); yxhuang@caf.ac.cn (Y.H.); wjy125616214@163.com (J.W.)

**Keywords:** water resistance, bamboo scrimber composite, bonding interface

## Abstract

As a new type of green environmental protection material for outdoor use, the water resistance of bamboo scrimber composite (BSC) is crucial—the primary reason for a decrease in water resistance being bonding interface failure. From a bonding interface structure perspective, the influence mechanism of the resin content and density on the water resistance of BSCs remains unknown. Therefore, in this study, BSCs were prepared using Moso bamboo and phenol-formaldehyde resin, and the changes in the macroscopic and microscopic bonding interfaces before and after 28-h water-resistance tests were observed and analyzed. The results showed that the water resistance of the BSC increased with increasing resin content, with higher thickness swelling rates (TSRs) observed at higher densities. Obvious cracks were found at the macroscopic interface after 28-h tests, with higher resin contents leading to fewer and smaller cracks. With increasing density, the longitudinal fissures due to defibering process decreased, having an effect on width swelling rates (WSRs). Furthermore, porosity measurements revealed changes in the microscopic bonding interface; the difference in porosity before and after testing (*D*-value) showed the same trend as water resistance. Generally, we conclude that the macroscopic and microscopic bonding interface structures are closely related to BSC water resistance.

## 1. Introduction

With the continued growth in green buildings and sustainable urban construction, it has become necessary to develop green, renewable biomaterials to replace conventional structural materials—which are usually high density or polluting materials—such as metals, ceramics, and polymer composites [1,2,3,4]. Moso bamboo (*Phyllostachys pubescens* Mazel) accounts for 67% of all bamboo forests in China, and is therefore used more extensively than other bamboo species [5]. The findings of previous studies have shown that Moso bamboo fibers offer superior mechanical properties compared to wood fibers [6]. To fully exploit high-strength bamboo fibers, a novel strategy to use a Moso bamboo scaffold as a matrix reinforced by phenol-formaldehyde (PF) resin has been developed. Bamboo scrimber composite (BSC)—a renewable, engineered material—consists of crushed bamboo bundles assembled along the grain direction after PF resin immersion and pressed into a dense block. With its multi-level and multi-scale structure, BSC has superb physicomechanical performance, high utilization ratios (over 90%), excellent carbon sequestration, and is widely used in various fields, including furniture, flooring, garden landscaping, construction, and wind turbine blades [7,8,9,10]. It also overcomes the defects of anisotropy and the inhomogeneous density of natural bamboo.

In recent years, most studies of BSCs have focused on their physical and mechanical properties. Kumar et al. investigated the effects of density on the mechanical and water absorption properties of BSCs prepared via cold-pressing and hot-curing. They found that the water absorption and all mechanical properties of BSCs—such as tensile strength, compression strength, shear strength, and flexural strength—increased with increasing density [11]. Sharma et al. compared the mechanical properties of two types of BSCs and laminated bamboo sheets [12]. Subsequently, Shangguan et al. investigated the mechanical properties and chemical composition of heat-treated BSCs [13]. Furthermore, Rao et al. found that, as the resin content increased, the water resistance of the BSCs increased, shear strength increased at first and then decreased, and compressive strength decreased [5]. Rao et al. also investigated the effects of different units and resin molecular weights on the mechanical performance and water resistance [14,15]. However, most reports have included only superficial discussions of the water resistance of BSCs, and few studies have been conducted on its influence mechanism.

Although qualified BSCs can be produced by most enterprises, some problems caused by poor water resistance still exist, such as cracking, fuzzing, and deformation under high-humidity conditions. Resin content and density are two vital factors that determine dimensional stability and water repellency [16]. As the resin content increases, the water resistance of composites increases gradually [17,18,19]. Rao et al. demonstrated the optimum resin content of BSC to be 20% [5]. Based on previous studies [17,20], density has a major influence on the water absorption properties of composites. For example, the mechanical properties increased with increasing density [21], whereas the water absorption was reduced [11,22]. Research on the water resistance of BSCs has focused primarily on performance but has not established the essential influences, especially on the bonding interface changes.

Improvement in water resistance can be achieved by ameliorating the compatibility of the bonding interface [23]. The fundamental reason for the decline in dimensional stability is the interface failure caused by uneven adhesive penetration and poor bonding [17,24,25,26]. Macroscopic interface failure refers to interlaminar cracking, which can be caused by weak gluing between two fluffed bamboo mats (FBMs). Microscopic interface failure refers to the springback and swelling deformation of the compressed cell cavity in a humid environment at a position with less effective bonding. Therefore, it is of vital importance to understand how cooperative coupling between the macroscopic and microscopic structures at the bonding interface affect the water resistance of BSCs. Meng et al. investigated the bonding interface structure via fluorescence microscopy, scanning electron microscopy coupled with energy-dispersive X-ray spectroscopy (SEM-EDX), and transmission electron microscopy (TEM). The results suggested that cured PF resin not only entered the cell lumina but also was deposited on the bamboo cell walls surface [23]. Yu et al. also confirmed that the cells in BSC were filled with PF resin through SEM and TEM observation [17]. However, few studies have been conducted on the response relationship between the bonding interface failure and water resistance of BSCs.

In addition, it is worth mentioning that researchers usually choose to increase the concentrations of PF resin (appropriately) to solve the problem that the FBMs cannot absorb adequate, predetermined adhesive volumes because of complete saturation during the impregnation process, which can result in experimental errors due to differences in the permeabilities of PF resins with inconsistent resin solid concentrations.

In this study, PF resin with the same solid concentration was used, with the procedure from dipping to drying being repeated several times until the stated dose had been reached. We examined the influence of the resin content and density on the water resistance of BSCs from an interface failure perspective. Furthermore, the macroscale and microscale bonding interface structures of untreated samples under different preparation conditions were observed using laser scanning microscopy (LSM) and SEM. The macroscopic interface failure morphology was observed after water-resistance tests, using ultra-depth electron microscopy (UDEM), the microscopic interface failure being reflected by changes in the porosity and pore-diameter distribution before and after the test.

## 2. Material and Methods

### 2.1. Materials

Four-year-old Moso bamboo (*Phyllostachys pubescens* Mazel) was obtained from Huzhou, Zhejiang Province, China. PF resin of 47.5 wt%, the viscosity of 40 cps, Mw of 919, and pH of 10.04 was supplied by the Beijing Dynea Chemical Industry Co., Ltd., Beijing, China. Safranine T (Macklin) and toluidine blue O (Macklin) were used as received.

### 2.2. Preparation of BSC

**Figure 1:**
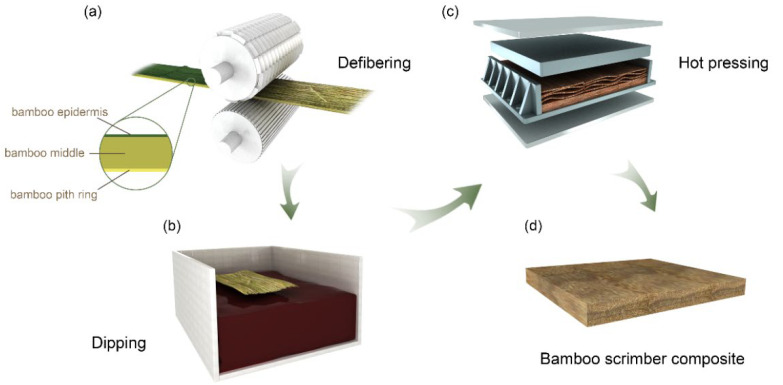
Schematic illustration of BSC preparation. Note: (**a**) Defibering; (**b**) Dipping into the PF resin; (**c**) Hot pressing; (**d**) BSC.

The method used to produce the BSC is shown in Figure 1. The bamboo was defibered using a crushing machine to obtain FBMs with a series of uniform linear cracks along the grain direction (Figure 1a). Bamboo is divided into three parts in the enlarged schematic diagram of a bamboo section: bamboo epidermis (BE), bamboo middle (BM), and bamboo pit ring (BPR), and some parts of the BS and BPR are removed during defibering. The side close to the BS in an FBM is referred to as “the outer surface”, while that close to the BPR is “the inner surface”. The FBMs were then dipped into the PF resin, which was diluted into 15 wt% solutions by adding distilled water (Figure 1b). After air-drying to 10% MC, the FBMs with target resin contents were assembled into the mold along the grain based on the desired density, as summarized in Table 1. After pressing in a hot-pressing machine (Carver 3925, CARVER Inc., Wabash, IL, USA) at 150 °C for 10 min and unloaded while cooling at 30–40 °C, BSCs of dimensions 300 × 120 × 10 mm (longitudinal × width × thickness) were obtained (Figure 1c,d). We screened the samples with density ranges less than ±0.02 g/cm^3^ from the set value and considered them to have the same density gradient. Finally, all BSCs were conditioned in a chamber at 20 ± 2 °C and relative humidity (RH) of 65 ± 5% for 2 weeks before being tested.

### 2.3. Characterizations

#### 2.3.1. Water Resistance

The water absorption rate (WAR), width swelling rate (WSR), and thickness swelling rate (TSR) of the BSC samples were determined based on the GB/T 40247-2021 standard. The 28-h cycle hydrothermal treatment included 4 h of immersion in boiling water at 100 ± 2 °C, 20 h of oven-drying at 63 ± 3 °C, and another 4 h of immersion in boiling water at 100 ± 2 °C. The sample surface was placed parallel to the horizontal plane in a water bath kettle. Four samples of dimensions 50 × 50 × 10 mm^3^ were tested in each case. Then, the weight and size changes were measured after carefully removing any excess water from the specimen surfaces. The WAR, WSR, and TSR were calculated using Equations (1)–(3):

(1) $$\mathrm{WAR}\left(\%\right)=\frac{m_{1}-m_{0}}{m_{0}}\times 100$$

(2) $$\mathrm{WSR}\left(\%\right)=\frac{b_{1}-b_{0}}{b_{0}}\times 100$$

(3) $$\mathrm{TSR}\left(\%\right)=\frac{t_{1}-t_{0}}{t_{0}}\times 100$$

where m_0_, b_0_, and t_0_ are the weight, width, and thickness of the pristine samples, respectively, and m_1_, b_1_, and t_1_ are the weight, width, and thickness of the 28-h cycle-treated samples, respectively.

Based on the experimental data of the water-resistance tests, the optimum process conditions of BSCs—resin content: 20%, density: 1.15 g/cm^3^ (vide infra)—were selected to determine the value of the other fixed variable when one of the variables changes. Therefore, BSC samples obtained under five conditions with different resin contents and densities, namely BSC-10-1.15, BSC-15-1.15, BSC-20-1.0, BSC-20-1.15, and BSC-20-1.3, where “BSC-x-y” refers to a resin content of x% and a density of y g/cm^3^, were prepared for the following tests.

#### 2.3.2. Morphological Characterizations

Cross-sections of BSC samples 10 × 10 × 10 mm in size, prepared using the five above-mentioned process conditions, were cut with a slicer (RM2245, LEICA, Wetzlar, Germany). To analyze the distribution of the PF resin through BSCs of different resin contents, LSM (LSM 980 with Airyscan 2, Zeiss, Oberkochen, Germany) was performed. Specimens were stained with 0.5% toluidine blue O prior to LSM testing to suppress the auto-fluorescence of the bamboo lignin. The cell deformation of the BSCs at different densities was examined using SEM (SU8020, Hitachi, Ltd., Tokyo, Japan). The failure morphologies of the cross-sectional surfaces at the macroscopic bonding interfaces of the BSC samples were observed by UDEM (VHX-6000, Keyence, Osaka, Japan) in transmission mode following completion of the 28-h cycle treatment.

#### 2.3.3. Mercury Intrusion Porosimetry (MIP)

MIP testing was performed using a Mercury Porosimeter (AutoPore V 9600, Micrometrics Inc., Norcross, GA, USA) in the 0.0007–420.5950 Mpa pressure range. Because mercury is a non-wetting fluid—which means it cannot penetrate a porous solid through capillary forces—we approximated bamboo as a bundle of small cylindrical capillaries to simplify the calculations [27]. The pore volume was obtained based on the quantity of mercury intruded; hence, the total internal volume could be calculated. In addition, based on the Washburn equation [28], the pore radius (*r*), porosity (*φ*), and aperture size distribution (*D(r)*) can be determined as follows:

(4) $$r=-\frac{2\gamma \cdot \cos\theta }{P}\;$$

(5) $$\varphi =\frac{V_{T}}{V_{S}}\;$$

(6) $$D\left(r\right)=\frac{P}{r}\frac{dV}{dP}\;$$

where *P* is the measured pressure, *γ* is the surface tension of mercury (0.48 N/m), *θ* =140° is the wetting angle of mercury, *V_T_* is the total volume of mercury, and *V*_S_ is the total sample volume.

In addition, in order to compare the change in porosity before and after the water resistance test, the porosity difference is defined as *D*-value (*D*), which is calculated by the following equation:

(7) $$D=\varphi_{1}-\varphi_{0}$$

where *φ*_0_ is the porosity of untreated BSC and *φ*_0_ is the porosity of BSC after 28-cycle treatment, respectively.

### 2.4. Statistical Analysis

To detect the difference in water resistance among the BSC samples prepared using different resin contents and densities, analysis of variance (ANOVA) was performed using SPSS (IBM SPSS software version 24, SPSS Inc., Chicago, IL, USA)—F being the outcome of the F test, which equals the ratio of the mean square between groups and within groups. Additionally, p can determine the significance of differences and represent probabilities under the corresponding F values, while defining 5% as the significance level (*p* < 0.05).

## 3. Results and Discussion

### 3.1. Dimensional Stability

**Figure 2:**
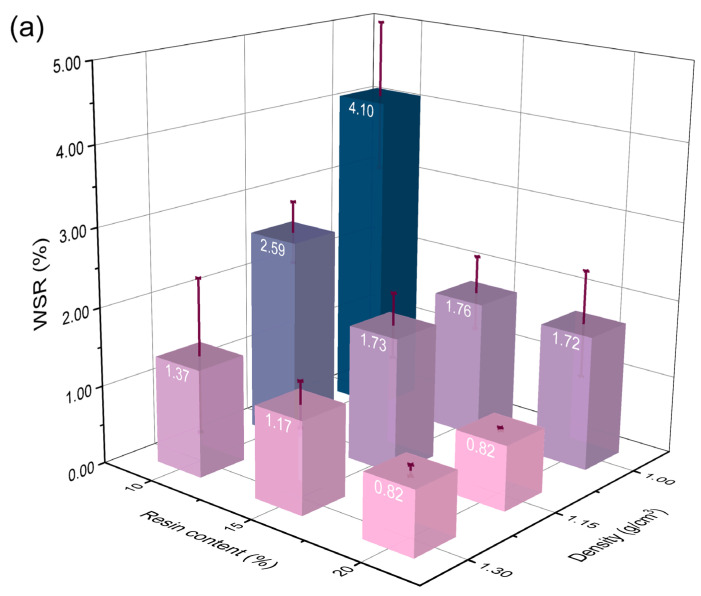

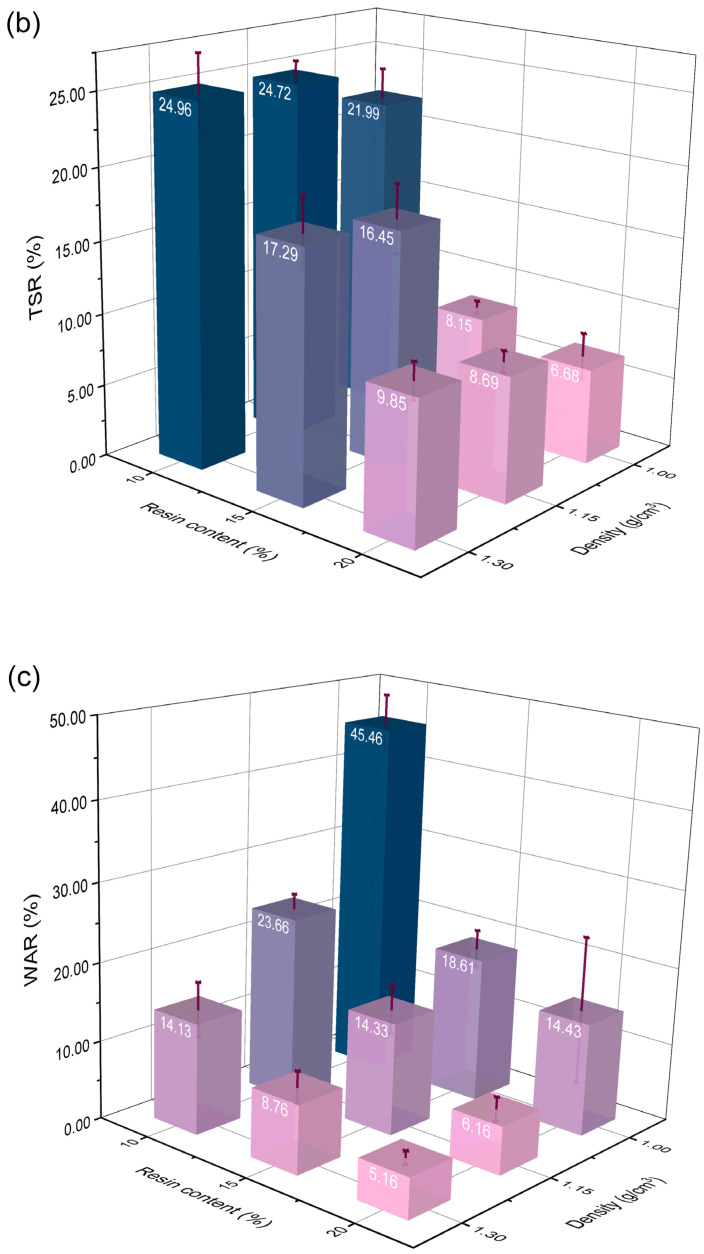
Water resistance of BSC samples including (**a**) WSR, (**b**) TSR, and (**c**) WAR.

Resin content and density are two main factors that affect BSC water resistance. Figure 2 shows the WSR, TSR, and WAR of the BSCs after 28-h cycle treatment. The WSR and WAR of BSC-20-1.3 were significantly lower than those of the other samples, which was the minimum value among the BSCs in this study. For BSC-20-1.0, the TSR was as low as 6.68%, which was the lowest value among the BSCs in this study. The high standard deviations of the WSR and WAR for BSC-20-1.0 were due to the high variability among the samples. At the set levels, it had the highest resin content (20%). Correspondingly, the percentage of bamboo in BSC-20-1.0 was the smallest with the same density, so it was more dispersed and had more voids in the lay-up process. The WSR and WAR of BSC-10-1.0 had high standard deviations, indicating that the lower resin content can also lead to higher variables due to uneven permeation of PF resin. Meanwhile, these findings are attributed to the fact that both BSC-10-1.0 and BSC-20-1.0 had the lowest hot-pressing pressure (smallest density) and the resin did not flow uniformly to all parts of the samples; thus, the performance gaps among BSC samples at 1.00 g/cm^3^ density were large.

As the resin content of the BSC increased, the WSR, TSR, and WAR gradually decreased during the water-soaking test. This is attributable to the fact that water can penetrate bamboo parenchymal cells, vessels, or intercellular spaces, and be absorbed through hydrogen bonds due to the many free hydroxyl groups in the bamboo cell wall [8,29]. Moreover, the impregnation of the PF resin increased after the defibering process. PF resin—which can penetrate the cell lumens, swell the cell wall, and form a rigid cross-linked hydrophobic network during curing—helps to improve the dimensional stability of bamboo by blocking the impregnation path of water [7,30,31,32]. Therefore, the ability of the network system to effectively prevent the hydroxyl groups of bamboo from interacting with water molecules is strengthened with an increase in the resin content.

With a density of 1.00 g/cm^3^, the WSR, TSR, and WAR of BSC-10-1.0 were 2.39, 3.29, and 3.15 times those of BSC-20-1.0, respectively. By contrast, when the density increased to 1.30 g/cm^3^, the WSR, TSR, and WAR of BSC-10-1.3 were 1.67, 2.53, and 2.74 times those of BSC-20-1.3, respectively. This phenomenon showed that the higher resin content BSCs had higher water resistance—consequently, 20% was selected as the optimum resin content condition. Moreover, a higher density could decrease the effect of resin content on the water resistance of BSCs. Based on the ANOVA results shown in Table 1, the resin content significantly affected all values of water resistance (p < 0.05).

The TSR—which represents the swelling of the bamboo cell wall plus the swelling caused by the compressive release—showed an upward trend with increasing BSC density after the water-soaking test (Figure 2b). The hydrothermal cycle treatment was more efficient or faster in inducing the TSR in higher-density BSCs. The TSR of BSC-20-1.3 was 47.46% higher than that of BSC-20-1.0. However, a high density had a positive effect on the WSR and WAR of the BSC. These findings could be ascribed to the closure of the water infiltration path due to the formation of interlocked structures between the dense cells and PF resin under high pressure [22]. Based on the ANOVA analysis (Table 1), the density showed clear effects on the WSR and WAR of BSCs (p < 0.05), except for the TSR (p *=* 0.291 > 0.05). At a resin content of 20%, the WSR and WAR of BSC-20-1.3 were 52.33% and 64.24% lower than BSC-20-1.0, respectively.

It should be noted that the water resistance of a material is good only when all three values (WSR, TSR, and WAR) are low. As mentioned above, 20% was chosen to be the optimum resin content. At 20% resin content, the WAR and WSR of BSC-20-1.3 were the lowest and the TSR of BSC-20-1.0 was the smallest. Considering all water resistance indicators, 1.15 g/cm^3^ (the middle value) was confirmed to be the best density. Therefore, the best process was that with 20% resin content and 1.15 g/cm^3^ density. Besides the samples under the optimum process (BSC-20-1.15), BSC-10-1.15, BSC-15-1.15, BSC-20-1.0, and BSC-20-1.3 were also selected for the subsequent characterization to study the effects of the resin content and density on the water resistance of BSCs. Moreover, the interaction between resin content and density had a significant influence on the WSR, TSR, and WAR (p < 0.05), which also revealed that they were not independent factors and had interactive effects on each other (Table 2).

### 3.2. Bonding Interface Morphology

**Figure 3:**
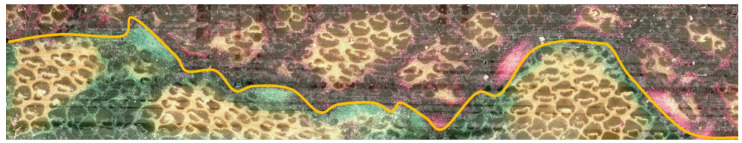
The comb-meshing structure of the macro bonding interface.

Two FBMs, separately dyed with Safranine T and Toluidine Blue O, were hot-pressed to prepare BSCs under the optimum process conditions. As shown in Figure 3, uneven dyeing outlined the shape of the bamboo fiber bundles, where the cracks generated by the defibering process were compressed and cross-linked with the PF resin. In addition, the yellow curve—that is, the boundary between the two FBMs after hot pressing—shows a comb-meshing structure. Compared with other bamboo/wood composites, this bonding structure is unique and stable and provides BSCs with higher strength and superior water resistance.

LSM can reveal the distribution of the PF resin—hence, BSC-10-1.15, BSC-15-1.15, and BSC-20-1.15 were selected for observation. The laser wavelengths were set to 405 nm (green channel) and 488 nm (red channel).

**Figure 4:**
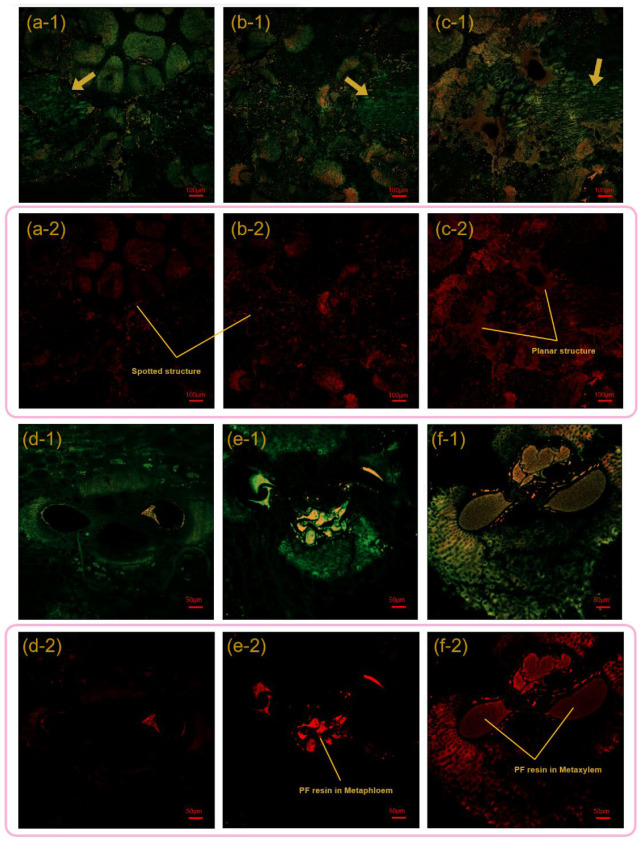
The LSM images of the macroscopic bonding interface and the cured PF resin trapped in the bamboo vascular tissue: (**a**,**d**) BSC-10-1.15, (**b**,**e**) BSC-15-1.15, (**c**,**f**) BSC-20-1.15. Note: 1 and 2 denote dual-channel superposition diagrams and single-channel diagrams, respectively.

In Figure 4, green represents lignin, while orange and red represent the PF resins. It is worth mentioning that orange is formed by the superposition of the red and green parts in the dual-channel mode. The yellow arrows refer to the residual parts of the BPR—that is, stone cells. In contrast to other bamboo composites, the macroscopic bonding interface of the BSC was in the form of point-to-plane bonding rather than linear [33,34]. However, when the resin content was less than 15%, the bonding interfaces were spotted structures (Figure 4(a-2,b-2)), with planar structures appearing at resin contents of up to 20% (Figure 4(c-2)). When the resin content increased from 10% to 20%, the area of the red part increased, which meant that the PF resin was better distributed (Figure 4a–c). It was also determined that the penetration of the PF resin could be affected by the resin content.

Although a few fiber cells were not completely shielded by the toluidine blue O, a general trend could still be observed (Figure 4). From Figure 4d–f, the distribution of PF resin in the vessels reflected the growth of resin content. Cured PF resin filled the metaphloem and metaxylem at 20% resin content (Figure 4(f-2)).

**Figure 5:**
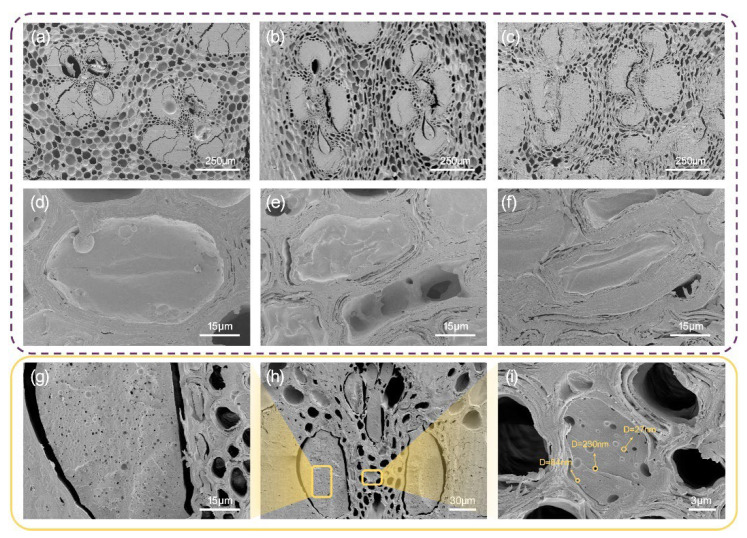
SEM micrographs of BSC-20-1.0 (**a**,**d**), BSC-20-1.15 (**b**,**e**), BSC-20-1.3 (**c**,**f**), and cured PF resin (**g**–**i**). Note: D denotes the diameter of the pore.

The parenchyma tissues and vessels of the FBMs were densified during hot pressing. As the density increased, the degree of deformation increased, according to the SEM diagrams shown in Figure 5. It could be seen that, when the density was less than 1.15 g/cm^3^, more PF resin remained in the lumens. At 1.30 g/cm^3^, the cell cavity and vessels were almost closed, causing large internal stress, with less PF resin present—which meant that the binding force of the PF resin on cell rebound would be reduced if the samples were in a hydrothermal environment, resulting in a decline in diameter stability. This inference is consistent with the previous water-resistance test results. In addition, many nanopores with diameters ranging from tens to hundreds of nanometers could be observed in the cured PF resin (Figure 5g–i).

### 3.3. Macroscopic Interface Failure Morphology

**Figure 6:**
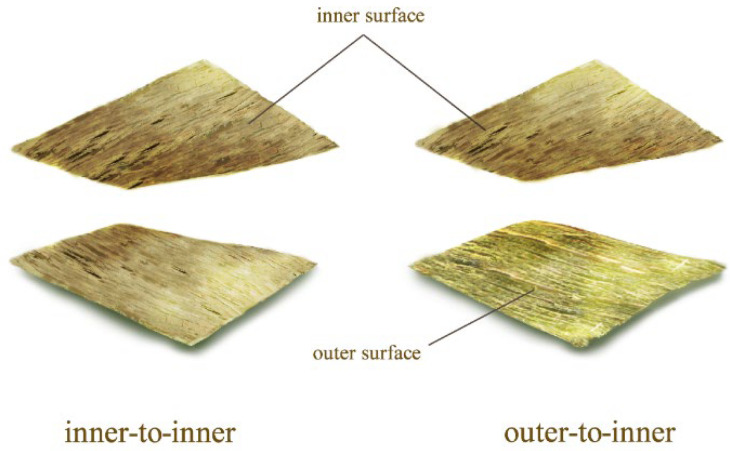
Two BSC assembly patterns.

**Figure 7:**
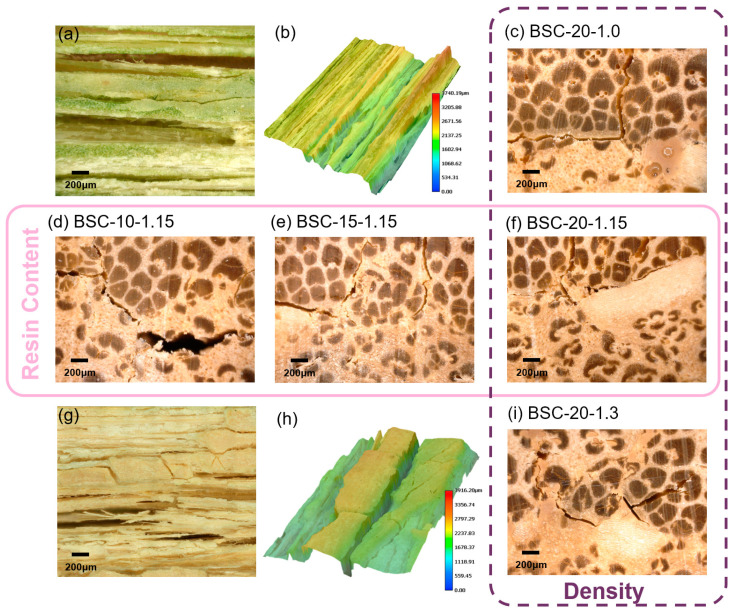
2D (**a**,**g**) and 3D (**b**,**h**) images of the outer (**a**,**b**) and inner (**g**,**h**) surfaces of the FBM and failure morphologies at the outer-to-inner bonding interfaces of the BSCs (**c**–**f**,**i**) after the 28-h cycle treatment. Note: The pink frame represents BSCs at various resin contents; the purple frame represents BSCs at various densities.

The FBM has two sides, namely the outer and inner surfaces, and two BSC assembly patterns (outer-to-inner and inner-to-inner) were observed (Figure 6). Figure 7a,g show the original 2D morphologies of the bamboo fiber bundles in the FBMs. Bamboo has no radial transfer structure that prevents the water-soluble PF resin from entering internal cells [35]. Consequently, a defibering process was adopted to create dot- and linear-shaped fissures that disrupt few fibers—which is not only conducive to the penetration of the adhesive but also maintains the outstanding strength of the natural bamboo. The brittle parts of the BE and BPR fell off easily during the defibering process. In the 3D images, the red-shaded areas correspond to the residual BE waxy layer (Figure 7b) and stone cells in the BPR (Figure 7h) of the bamboo after mechanical fluffing. In addition, the maximum linear-shaped crack depths of the outer and inner surfaces were determined to be 3740.19 and 3916.20 μm, respectively, which were sufficient to open the internal impregnation channels.

**Figure 8:**
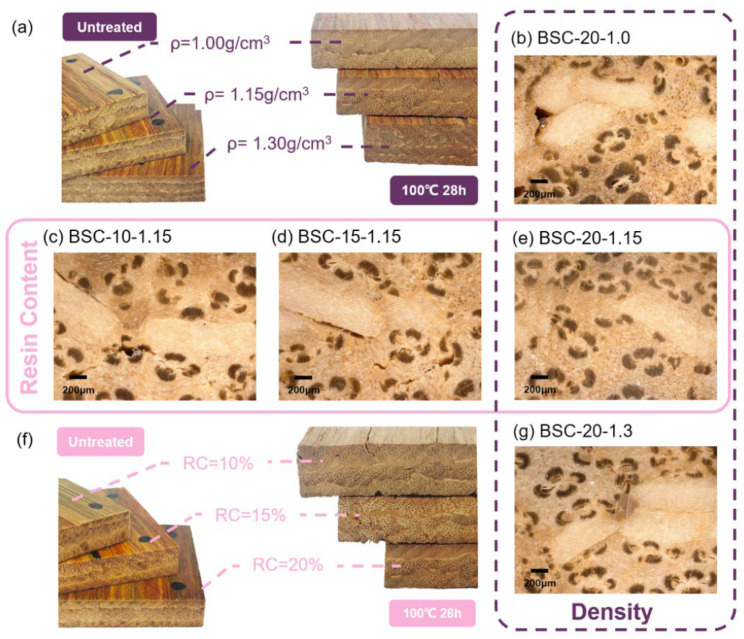
Images of BSC samples at various density (**a**) and resin content (**f**) conditions before and after the water-resistance test and failure morphologies at the inner-to-inner bonding interfaces of the BSCs (**b**–**e**,**g**) after the 28-h cycle treatment. Note: The pink frame represents BSCs at various resin contents; the purple frame represents BSCs at various densities.

Compared to specimens of different densities (Figure 8a), it can be seen from the photos that changes in the thickness of BSCs with different resin contents before and after the 28-h cycle treatment were more substantial (Figure 8f), indicating that the resin content had a greater impact on the TSR than the density. This was also proven by the previous ANOVA results. Furthermore, the BSC-20-1.3 sample had more cracks in its macroscopic appearance than BSC-20-1.0, after the 28-h cycle treatment (Figure 8a).

To explore the effect of the resin content and density on the water resistance, the failure area of the macroscopic bonding interface of the BSCs was observed along a transverse section. By means of UDEM images, we established that the outer-to-inner bonding interfaces were always damaged after treatment when the density was raised. However, increasing the resin content contributed to clear improvements (Figure 7). Destruction of the macroscopic bonding interface mostly occurred where the BE and BPR remained. The width and number of transverse cracks decreased with increasing resin content (Figure 7d–f). The TSR showed the same trend with changing resin content (Figure 2b). In the case of BSC-20-1.3 (Figure 7i), new thin cracks in the transverse direction appeared after the water-resistance test as a result of the stress stored via high compression, which led to a larger springback [36], but generally speaking, no obvious transverse fracture trend was observed as the density was varied. However, the longitudinal cracks due to the defibering process decreased with an increase in density (Figure 7c,f,i) because the PF resin increased its fluidity under the action of high temperature and high pressure, making it easier to penetrate these large fissures. This also explains why WSR correlates negatively with density, as shown in Figure 2a. There were visible cracks between the inner and inner surfaces of BSC-10-1.15 and BSC-15-1.15, whereas there were no fractures in the others (Figure 8). In general, the thickness modification depends on the bonding strength of the outer-to-inner interface. Overall, at either bonding interface, there was an inverse correlation between the resin content and the number of cracks, indicating that the increase in PF resin adhesive improved the interfacial bonding strength.

### 3.4. Analysis of Porosity

**Figure 9:**
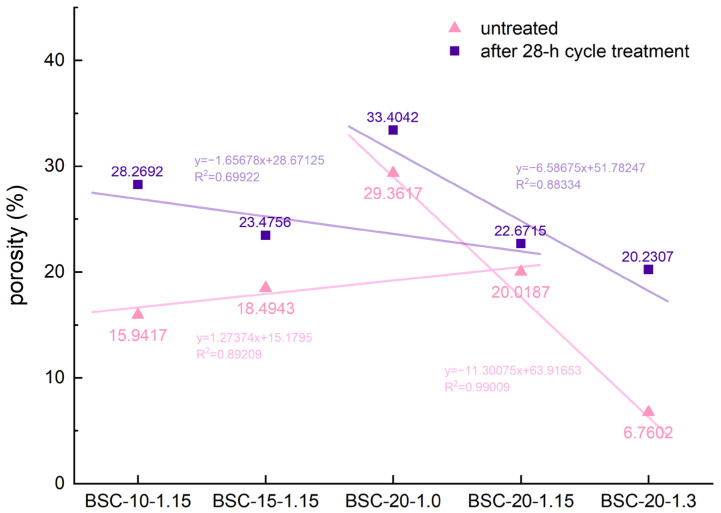
Porosity of the BSC samples before and after 28-h cycle treatment.

It was difficult to directly observe the failure morphology of the microscopic bonding interface; therefore, the pore information was measured using MIP tests to indirectly reflect the interface. Prior to analyzing the pore size distribution, the porosity of the BSC samples under different preparation conditions should first be discussed. Figure 9 shows the relationship between the resin content/density and porosity of the BSCs before and after the 28-h cycle treatment. As observed from the data of untreated and treated BSCs, the increase in density led to a decrease in porosity owing to the compression and closure of partial lumens, which was confirmed by previous morphological observations. BSC *D*-values at various densities (Table 3) were calculated, which revealed that BSC-20-1.3 has the largest *D*-value, consistent with the previous conclusion about the correlation between density and TSR, which indicated that changes in the microscopic bonding interface caused by the hydrothermal treatment might be the reason for the change in the TSR. Furthermore, the porosity of the untreated samples exhibited a positive correlation with the resin content, whereas the porosity of the treated group was negatively correlated. The *D*-value of BSC-20-1.15 was considerably less than that of BSC-10-1.15; consequently, the parts of the pores that increased due to growth in resin content did not change much under hydrothermal action. Combined with the above morphological analysis, we speculate that the increased parts of pores are formed via the curing of the PF resin. The *D*-value of BSC-20-1.15 was the smallest of all samples, consistent with the best conditions selected using the water-resistance test mentioned above. This also confirmed that the failure of the microscopic bonding interface was closely related to water resistance.

**Figure 10:**
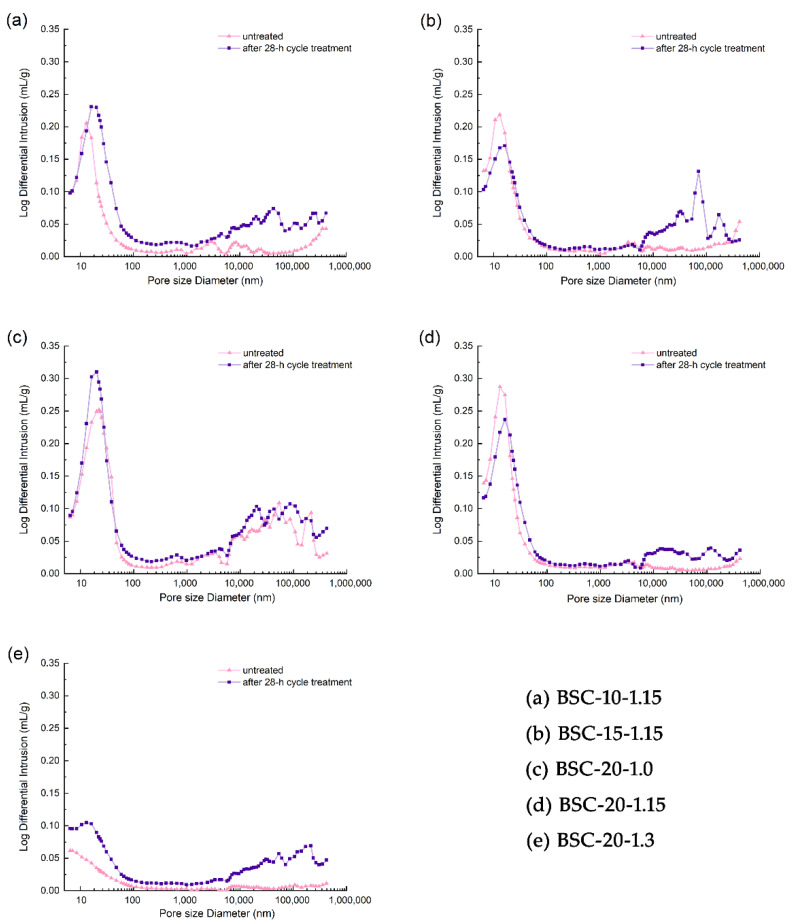
Pore size distribution of the BSC samples before and after 28-h cycle treatment. Note: (**a**) BSC-10-1.15; (**b**) BSC-15-1.15; (**c**) BSC-20-1.0; (**d**) BSC-20-1.15; (**e**) BSC-20-1.3.

Typical MIP curves for the log differential intrusion versus pore size diameter for the BSCs are shown in Figure 10. Based on the peak positions, it can be concluded that the pore distribution in the BSCs could be divided into two parts—that is, pores of diameters of 10–100 nm and those >1000 nm. The bamboo structure consisted of parenchyma, fibers, and vessel cells. These component cells had lumens with diameters in the range of 21–40 μm [37,38] for parenchyma, 0.26–24.96 μm for fibers [39,40], and 40.17–259.91 μm for vessels [21].

Pores with diameters ranging from 10–100 nm could be pit-membrane voids or other small voids [41]. With an increase in the resin content, the number of pores (10–100 nm) in the untreated samples increased (Figure 10a,b,d). After the 28-h cycle treatment, there were fewer nanopores (10–100 nm) of BSC-15-1.15 and BSC-20-1.15 than those of the untreated group, which might have been due to the PF resin filling the void space of the pits. In addition, it also showed that some nanopores produced due to the increase in resin content had excellent hydrophobicity. This might be because of the pores formed by water evaporation in the PF resin during curing, further confirming earlier speculation. In contrast to BSC-20-1.0 and BSC-20-1.3, BSC-20-1.15 exhibited fewer pores (10–100 nm) before treatment than after treatment (Figure 10c–e), suggesting that the PF resin could fully penetrate and fill the nanopores under the proper stress only at a density of 1.15 g/cm^3^.

From Figure 10, it can be seen that the 28-h cycle treatment led to an increase in the number of pores of diameters greater than 1000 nm. Micron pores (diameters greater than 1000 nm) contain cavities of various cells, cell gaps, cracks generated by defibering, gaps between FBMs, etc. The micron pores (>1000 nm) of BSC-20-1.15 were smaller than those of BSC-10-1.15. Compared with the pore size distribution before and after the 28-h cycle treatment, the distribution of micron pores (>1000 nm) in the samples after the 28-h cycle treatment decreased as the resin content increased (Figure 10a,b,d)—that is, as the resin content increased, the permeability of the PF resin increased, making it easier for it to enter the larger pores. Meanwhile, with the increase in density, the micron pore distribution of untreated BSC decreased considerably, caused by the higher compaction ratios (Figure 10c–e). Based on the untreated group, the changes in the micron pores in the samples after 28-h of cycle treatment increased as the density increased, revealing that excessive pressure weakened the binding force of the PF on micron pores, which rebounded more during the water-resistance test.

## 4. Conclusions

In this study, the water resistance and macroscopic and microscopic bonding interface characterization of BSCs with different resin contents (10%, 15%, and 20%) and densities (1.00 g/cm^3^, 1.15 g/cm^3^, and 1.30 g/cm^3^) were investigated. With an increase in density, the TSR increased, while the WSR and WAR decreased. At the same time, the resin content was inversely proportional to the three water-resistance indices of the BSCs. The BSC water resistance at a resin content of 20% and density of 1.15 g/cm^3^ was the best.

It could be concluded that there were nonlinear and point-to-plane structures in the macroscopic bonding interface, and that the PF resin was more evenly and widely distributed with an increase in resin content. Moreover, as the density increased, the compression of the cell cavity and vessels increased.

To analyze the influence mechanism of the resin content and density on the water resistance of the BSCs from a bonding interface perspective, the failure morphology of the macroscopic bonding interface after the water-resistance test was observed, which showed that the failure of the outer-to-inner interface had a more obvious effect on water resistance than that of the inner-to-inner interface. The transverse and longitudinal fissures at the macroscopic bonding interfaces on the cross-sections of the BSCs decreased considerably with increasing resin content, which was consistent with the trends of WSR and TSR. With increasing density, only longitudinal fissures due to defibering decreased, which affected the WSR. In conclusion, the influence of the resin content on all water-resistance indices of the BSCs could be reflected by the macroscopic interface failure, which could only explain the dimensional changes in the width direction concerning the density factor.

The microscopic bonding interface failure was measured using a MIP test, and the changes in porosity and pore distribution before and after 28-h cycle treatment were compared. As the resin content increased, the PF resin filled more nano- and micron-sized pores. The *D*-value was the smallest when the resin content was up to 20%. When the density increased, the porosities of the untreated and treated samples decreased. The *D*-value at a density of 1.30 g/cm^3^ was the largest. The microscopic bonding interface structure affected all water resistance indicators, with the TSR being more influenced than the other indicators. Therefore, the failure of the microscopic bonding interface could suggest the influence mechanism of the two factors on the BSC water resistance.

Water resistance plays an important role in BSCs and depends on the lifespan of the related products. Research on the water repellency and dimensional stability of BSCs from a bonding interface structure perspective is innovative and has theoretical significance for guiding performance improvements. Further research should be conducted to investigate novel methods that could be used to determine the influence mechanism of water resistance from a bonding interface perspective at other scales, such as ultramicroscopic and molecular scales.

## Figures and Tables

**Table 1:** BSC preparation conditions.

Sample	Preparation Conditions	
	Resin Content (%)	Density (g/cm^3^)
BSC-10-1.0	10	1.00
BSC-10-1.15	10	1.15
BSC-10-1.3	10	1.30
BSC-15-1.0	15	1.00
BSC-15-1.15	15	1.15
BSC-15-1.3	15	1.30
BSC-20-1.0	20	1.00
BSC-20-1.15	20	1.15
BSC-20-1.3	20	1.30

**Table 2:** Water-resistance data analyzed by ANOVA.

Factor	WSR		TSR		WAR	
	F	p	F	p	F	p
resin content	7.057	0.004	55.425	0.000	8.770	0.001
density	4.881	0.017	1.301	0.291	6.260	0.006
interaction	3.293	0.034	3.912	0.019	9.395	0.000

**Table 3:** *D*-values of BSC samples.

Sample	*D*-Value/%
BSC-10-1.15	12.3275
BSC-15-1.15	4.9813
BSC-20-1.0	4.0425
BSC-20-1.15	2.6535
BSC-20-1.3	13.4705

## Data Availability

The data presented in this study are available on request from the corresponding author.
